# Multi-domain structures in spheroidal Co nanoparticles

**DOI:** 10.1038/s41598-020-67173-5

**Published:** 2020-06-23

**Authors:** N. A. Usov, M. S. Nesmeyanov

**Affiliations:** 1 National University of Science and Technology «MISiS», 119049 Moscow, Russia; 2 Pushkov Institute of Terrestrial Magnetism, Ionosphere and Radio Wave Propagation, Russian Academy of Sciences, (IZMIRAN), 108840 Troitsk Moscow, Russia

**Keywords:** Materials science, Nanoscience and technology, Physics

## Abstract

The structure of multi-domain micromagnetic states in hcp cobalt nanoparticles of spheroidal shape has been studied using numerical simulation in the range of diameters 20–200 nm. The single-domain diameters of the particles are determined depending on their aspect ratio. The complicated vortex structure of domain walls for two- and three-domain micromagnetic configurations is investigated. It has been shown that three domain states are actually strongly deformed two vortex states. In hcp cobalt particles of sufficiently large sizes two types of three-domain micromagnetic states with close total energies have been obtained. They differ in different magnetization directions of the exchange cores of the vortex domain walls. The remanent magnetization of particles has been calculated for two- and three-domain micromagnetic states. The single-domain diameters of fcc cobalt nanoparticles with cubic type of magnetic anisotropy were also calculated.

## Introduction

Cobalt nanoparticles attract substantial interest due to their technological significance^1^. They were also the first magnetic nanoparticles extensively studied theoretically^2–5^. However, the theory of small magnetic particles developed by Kittel^2^, Stoner and Wohlfarth^3^, Brown^4^, and Aharoni^5^ is only capable to describe the magnetic properties of single-domain nanoparticles. Meanwhile, magnetic nanoparticles of submicron sizes are often found in practice. For example, the assemblies of magnetic nanoparticles with a wide size distribution are frequently obtained in chemical synthesis^6^, so that the sizes of some particles may exceed the corresponding single domain diameter. Particles of sufficiently large sizes can arise during prolonged annealing of thin-film samples^7^, they can be created as a result of implantation process^8^, etc. It is worth noting that the magnetic properties of submicron-sized nanoparticles play an important role in paleo- magnetism^9^.

The magnetic properties of nanoparticles in inhomogeneous micromagnetic states differ significantly from those of single-domain ones^1,4,5^. However, the characteristics of nanoparticles of sufficiently large sizes are still poorly investigated. In particular, the dependence of the single-domain diameter of a spheroidal cobalt nanoparticle on its aspect ratio is unknown. The upper and lower analytical estimates for the single-domain diameter of magnetic nanoparticle given by Brown^10^ are not always close enough. Therefore, in most cases a detailed numerical simulation is necessary to determine the single-domain diameter of particles of various magnetic materials.

At present, domain structures in submicron cobalt nanoparticles and cobalt nanowires are experimentally studied using magnetic force microscopy^11,12^. In addition, modern electronic holography technique^13–17^ allows to study experimentally inhomogeneous micromagnetic distributions in magnetic nanoparticles and nanowires. This revives interest in detailed micromagnetic calculations of inhomogeneous magnetization distributions in nanoparticles of submicron sizes. Some practical applications of submicron nanoparticles are also probable. For example, it has recently been suggested^18^ to use nanoparticles in vortex micromagnetic states in magnetic nanoparticle hyperthermia.

The first simple variational estimate of the single domain diameter for a spherical cobalt nanoparticle was given by Kittel^2^ many years ago. Then, Stapper^19^ computed a one-dimensional magnetization distribution in a cobalt sphere with uniaxial anisotropy subdividing the particle into a large number of thin parallel slices. He showed that the energy of the two-domain state became lower than that of the uniform magnetization if the diameter of the sphere exceeded a critical value, *D*_*с*0_ = 76.0 нм. Aharoni and Jakubovics^20^ carried out the two-dimensional calculation of the magnetization distribution in a cobalt sphere under the restriction of cylindrical symmetry. For spherical cobalt particle they found that the cylindrically symmetric magnetization distribution with two domains separated by the cylindrical domain wall had lower energy than the one of Stapper. The single domain diameter of hcp cobalt nanoparticle was determined^20^ to be *D*_*с*0_ = 68.2 нм. Similar calculations were carried out also^21^ for a nanoparticle with cubic magnetic anisotropy.

However, both one- and two-dimensional micromagnetic calculations significantly limit the possible types of inhomogeneous micromagnetic distributions admissible in a three-dimensional magnetic nanoparticle. Significant progress in numerical modeling of inhomogeneous micromagnetic states was achieved after creating a numerical scheme for three-dimensional micromagnetic calculations^22–24^. Using the 3D numerical scheme the vortex magnetization distribution was first constructed in a magnetically soft cylindrical nanoparticle^25^. A characteristic feature of vortex distribution is the so called “exchange magnetization core”, i.e. relatively small region near the vortex center where the unit magnetization vector rotates rapidly to the direction perpendicular to the overall vortex magnetization outside the vortex center. This behavior of the unit magnetization vector excludes the magnetization singularity in the vortex center. Later it was realized that the vortex distributions are typical inhomogeneous micromagnetic states in magnetically soft nanoparticles of a rather arbitrary shape, such as a cube^26–28^, flat tablets^29–34^, spheres and ellipsoids^35–38^. It is now well known that the vortex magnetization distribution has as a rule the lowest total energy both in soft magnetic nanoparticles with magnetic anisotropy constant *K* small compared to the square of the saturation magnetization *M*_*s*_, *K* ≪ *M*_*s*_^2^, as well as for particles with moderate anisotropy constant value, *K* ~ *M*_*s*_^2^, such as hcp cobalt.

In this paper the two and three domain states in cobalt hcp nanoparticles are studied in detail using a numerical solution of the dynamic Landau – Lifshitz – Gilbert equation^4,5,38^. The complex vortex structure of domain walls separating two and three domain states is revealed. It is shown that in hcp cobalt nanoparticles there are two types of three domain states with close total energy. They differ in the direction of magnetization of the exchange cores of the vortex domain walls. The areas of existence of two and three domain states in hcp cobalt nanoparticles are determined, and the remanent magnetizations of particles in these states are obtained.

For completeness, inhomogeneous magnetization distributions in spheroidal cobalt fcc nanoparticles with cubic type of magnetic crystallographic anisotropy are also studied. These nanoparticles are often present^39^ in experimentally studied cobalt nanoparticle assemblies.

## Results and Discussion

### hcp cobalt

The following magnetic parameters were used in the numerical simulation of the magnetization distributions in hcp cobalt nanoparticles with a uniaxial type of magnetic anisotropy: saturation magnetization *M*_*s*_ = 1400 emu/cm^3^, uniaxial magnetic anisotropy constant *K* = 4.3 × 10^6^ erg/cm^3^, exchange constant C = 2 A = 2.6 × 10^−6^ erg/cm^1,40,41^. Stationary magnetization distributions in spheroidal nanoparticles with transverse and longitudinal diameters *D* and *D*_*z*_, respectively, were obtained by solving the dynamic Landau – Lifshitz – Gilbert (LLG) equation. The easy axis of magnetic anisotropy is assumed to be parallel to the particle axis of symmetry, which is directed along the Z axis of Cartesian coordinates. The magnetization distribution in cobalt nanoparticles is approximated by small cubic elements with a size *b* = 1–2 nm, which is much shorter than the exchange length in this material, ${L}_{ex}=\sqrt{A}/{M}_{s}$= 8.1 nm. The total number of numerical cells for particles of large diameters is about 3.6×10^5^. The inhomogeneous micromagnetic states are studied for cobalt nanoparticles with aspect ratios 0.5 ≤ *D*_*z*_/*D* ≤ 2.0 in the range of transverse particle diameters 20 ≤ *D* ≤ 200 nm.

#### Two domain configuration

**Figure 1:**
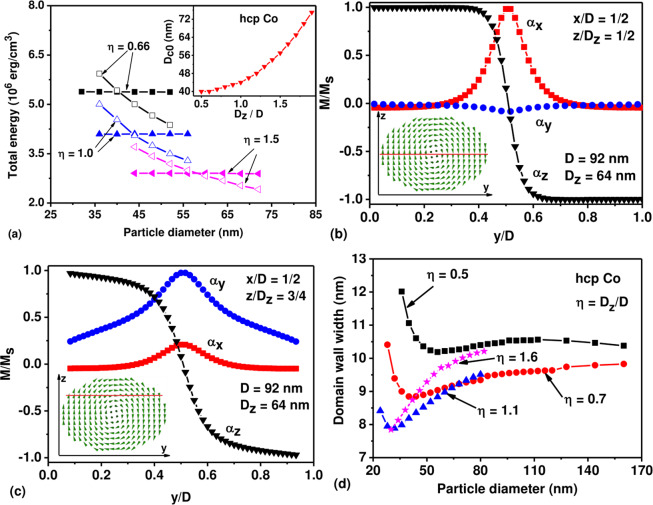
(**a**) Energy diagram of uniformly magnetized (filled symbols) and transverse vortex states (empty symbols) in cobalt hcp nanoparticles with different aspect ratios *η*. The inset in (**a**) shows the single domain diameter as a function of particle aspect ratio. (**b,c**) The structure of the vortex domain wall separating oppositely magnetized domains in a particle with sizes *D* = 92 nm and *D*_*z*_ = 64 nm for different sections of the nanoparticle shown schematically in the insets. (**e**) The width of the domain wall for two domain state depending on the aspect ratio and transverse diameter of cobalt hcp nanoparticle.

Fig. 1a shows the energy diagram of stationary micromagnetic states in cobalt hcp nanoparticles with various aspect ratios, *η* = *D*_*z*_/*D*, near the particle single domain diameter, where a uniform magnetization competes in energy with the transverse vortex state. The axis of the transverse vortex points perpendicular to the particle easy anisotropy axis. It is well known^4,5^ that the total energy of uniform magnetization in a particle of an ideal ellipsoidal shape does not depend on the particle size. On the other hand, as Fig. 1a shows for a given particle aspect ratio the total energy of the vortex decreases sharply as a function of particle diameter.

The intersection of the corresponding energy curves in Fig. 1a determines the transverse single domain diameter of cobalt hcp nanoparticle, *D*_*с*0_. According to Brown’s definition^4,10^, the single domain diameter of magnetic nanoparticle, *D*_*c*0_, is a size below which the uniform magnetization has the lowest total energy among all other stable magnetization distributions. For example, as Fig. 1a shows, for spherical nanoparticle, *η* = 1, the transverse vortex remains stable in some range of diameters *D* < *D*_*c*0_ = 45 nm. However its total energy in this region is higher than that of uniform magnetization. Therefore, the transverse vortex is metastable state at *D* < *D*_*c*0_. Similarly, uniform magnetization remains stable in some range of diameters *D* > *D*_*c*0_, where its total energy is higher than that of transverse vortex. Thus, uniform magnetization is metastable state at *D* > *D*_*c*0_.

According to Fig. 1a the single domain diameter depends on the particle aspect ratio. This dependence is shown in the inset in Fig. 1a. One can see that the single domain diameter of cobalt hcp nanoparticle increases significantly, from *D*_*с*0_ = 39.7 nm to *D*_*с*0_ = 74.2 nm, when particle aspect ratio changes in the range *η* = 0.5–2.0. Interestingly, with increasing particle size the transverse vortex state is strongly deformed and resembles the two-domain state predicted by Stapper^19^. However, 3D numerical simulation shows that unlike Stapper’s one-dimensional calculations the domain wall separating nearly oppositely magnetized domains retains a complex vortex structure. As an example, Fig. 1b,c show the structure of the vortex domain wall in an oblate cobalt nanoparticle with sizes *D* = 92 nm and *D*_*z*_ = 64 nm, respectively. In this particular case the exchange core of the vortex is magnetized in the positive direction of the *X* axis. Figure 1b shows the components of the unit magnetization vector *α*_*x*_, *α*_*y*_, and *α*_*z*_ depending on the coordinate *y*, 0 ≤ *y* ≤ *D*, along the central particle diameter, *x* = *D*/2, *z* = *D*_*z*_/2. As can be seen in Fig. 1b, the *α*_*z*_ component of the unit magnetization vector takes values close to ±1 in the left and right half of the particle, respectively. The *α*_*z*_ component changes sign within the exchange core of the vortex, *y* ≈ *D*/2, where *α*_*x*_ component approaches the value *α*_*x*_ ≈ 1. The *α*_*y*_ component is close to zero everywhere along the line considered.

In Fig. 1c the unit magnetization vector components are shown along the line *x* = *D*/2, *z* = *D*_*z*_/4. In this case the distribution of magnetization is more complex. But as before, the *α*_*z*_ component is large and positive within the left domain, and negative in the right domain. Thus, in the bulk of the particle the unit magnetization vector is approximately parallel to the easy anisotropy axis direction. This explains why the axis of the vortex in hcp cobalt nanoparticle lays perpendicular to the easy anisotropy axis. If the vortex core were magnetized along the easy anisotropy axis, the magnetization in the main particle volume, outside the exchange core, would be directed perpendicular to the easy anisotropy axis. The magnetic anisotropy energy for such magnetization distribution would be high making this state energetically unfavorable.The distribution of magnetization along the central particle diameter, perpendicular to the axis of the vortex, is used in this work to determine the characteristic width of the vortex domain wall. The domain wall width is determined in a usual manner^1^, as the thickness of a wall with a constant value of the derivative *dα*_*z*_/*dy* equal to that at the center of the wall. The characteristic width of the domain wall for particles with different aspect ratios is shown in Fig. 1d as a function of transverse particle diameter. Evidently, the domain wall width becomes approximately constant for sufficiently large particle diameters. Therefore, the ratio of the domain wall volume to the volume of the particle decreases with increasing transverse particle diameter.

The 1D calculations performed by Stapper for cobalt hcp nanoparticle^19^ are currently of historical interest only. Stapper’s hypothesis about the existence of a flat domain wall separating uniformly magnetized domains turned out to be unrealistic. In a cobalt hcp nanoparticle the flat domain wall is energetically unfavorable; the true domain wall has vortex structure. 1D calculations also lead to a significantly overestimated single-domain diameter of spherical cobalt nanoparticle. According to Stapper’s calculations it was determined to be *D*_*с*0_ = 76.0 nm. However, as Fig. 1a shows the true value of the single-domain diameter of spherical cobalt nanoparticle is much smaller, *D*_*с*0_ = 43.6 nm.

#### Three domain states

**Figure 2:**
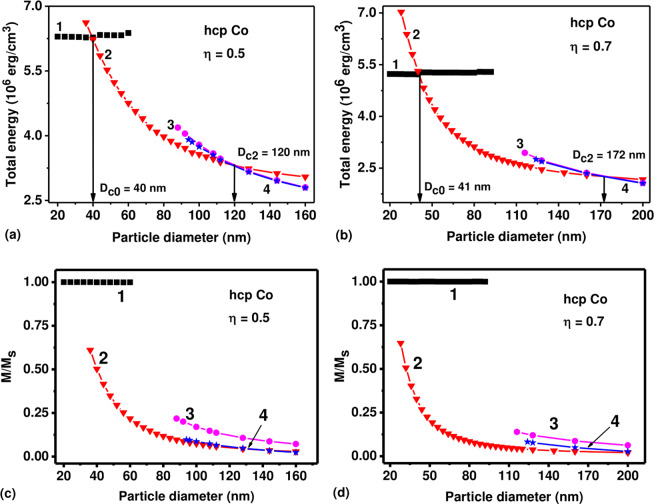
Energy diagrams of stationary micromagnetic states in cobalt nanoparticles with a large transverse diameter and various aspect ratios: (**a**) *η* = 0.5; (**b**) *η* = 0.7; (**c**), (**d**) the reduced magnetic moments of the particles depending on their transverse diameter. The notation of the curves in Fig. 2: (1) single-domain state; (2) transverse vortex state; (3), (4) two vortex states with identical and opposite directions of the exchange cores of the vortex domain walls, respectively.

The multi-domain micromagnetic configurations appear in cobalt hcp nanoparticles of sufficiently large sizes. These states determine the magnetic properties of submicron-sized cobalt nanoparticles and deserve a detailed study. Since reliable numerical simulation of the magnetization distributions in large nanoparticles requires significant computer resources, the calculations of multi- domain micromagnetic configurations in cobalt hcp nanoparticles are performed in this work in a limited range of transverse particle diameters, *D* ≤ 200 nm. Here two types of three-domain magnetization distributions that arise from strongly deformed two vortex micromagnetic states were discovered. For the first type of the three-domain state the exchange cores of vortices are magnetized identically, whereas for the second type they magnetized in opposite directions. Fig. 2 shows the energy diagrams of stationary magnetization distributions for oblate cobalt hcp nanoparticles with aspect ratios *η* = 0.5 and 0.7 in the range of transverse diameters 20 ≤ *D* ≤ 200 nm. According to Fig. 2, the single-domain diameters of oblate cobalt nanoparticles with aspect ratios *η* = 0.5 and 0.7 are given by *D*_*c*0_ = 40, and 41 nm, respectively. Further, three domain configuration becomes energetically favorable with respect to two domain one for the transverse particle diameter *D* > *D*_*c*2_, where *D*_*c*2_ = 120 nm for *η* = 0.5 and *D*_*c*2_ = 172 nm for *η* = 0.7, respectively. On the other hand, the three domain states are stable in the range of diameters *D* > 88 nm for *η* = 0.5, and in the range *D* > 116 nm for particles with aspect ratio *η* = 0.7, respectively.

**Figure 3:**
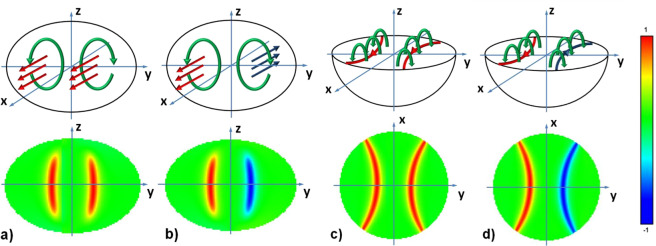
Two types of three domain micromagnetic states in cobalt nanoparticles with a sufficiently large transverse diameter. The upper panels schematically show the distribution of magnetization in different diametral sections of the particle, the lower panels give the distribution of the *α*_*x*_ component of the unit magnetization vector in the corresponding sections for the particle with *D* = 200 nm and aspect ratio *η* = 0.7. (**a**,**b**) the diametral cross section of the nanoparticle in the YZ plane for the case of vortex domain walls with similar and oppositely magnetized exchange cores, respectively; (**c**,**d**) the same for the case of diametral cross sections of nanoparticle in the XY plane.

The structure of three domain micromagnetic configurations in nanoparticles with a sufficiently large transverse diameter is shown schematically in the upper panels of Fig. 3. In fact, the three domain micromagnetic states are strongly deformed two vortex states. The bottom panels in Fig. 3 show the distribution of *α*_*x*_ component of the unit magnetization vector obtained numerically in the corresponding diametral sections of the nanoparticle. As can be seen in Fig. 3, the three domain states found differ in the various magnetization directions of the exchange cores of the vortex domain walls. But as Fig. 2a,b show, despite the difference in the magnetization distribution within the vortex domain walls the total energies of the two types of three domain states are very close.

**Figure 4:**
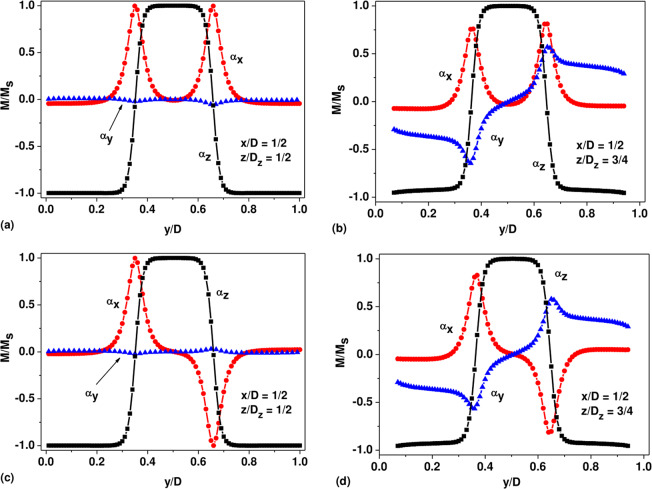
The structure of the vortex domain walls in a cobalt nanoparticle with sizes *D* = 200 nm, *D*_*z*_ = 140 nm for two types of three domain configurations: (**a**,**b**) the exchange cores of the vortex domain walls are magnetized similarly; (**c**,**d**) the exchange cores are magnetized oppositely.

Figure 4a,b show in detail the magnetization distribution in the nanoparticle with diameters *D* = 200 nm, *D*_*z*_ = 140 nm for three domain state with exchange cores of vortex domain walls magnetized along the positive direction of the *X* axis. Figure 4a shows the components of the unit magnetization vector depending on the coordinate *y*, 0 ≤ *y* ≤ *D*, along the central particle diameter, *x* = *D*/2, *z* = *D*_*z*_/2. In Fig. 4b, the magnetization distribution for the same micromagnetic state is shown as a function of the *y* coordinate along the line *x* = *D*/2, *z* = 3*D*_*z*_/4. The vortex structure of the domain walls separating oppositely magnetized domains is clearly visible in Fig. 4a,b.

As Fig. 4a shows, at the centers of the domain walls the unit magnetization vector component *α*_*x*_ = 1, whereas the other components are close to zero. At the same time, outside the domain boundaries the component *α*_*z*_ takes values close to −1 for the side domains and close to +1 for the central domain, while the other components are small. Consequently, in the bulk of the cobalt nanoparticle, the unit magnetization vector is approximately parallel to the direction of the easy anisotropy axis. This leads to a significant decrease in the magnetic anisotropy energy of the particle. As Fig. 4b shows, along the line *x* = *D*/2, *z* = 3*D*_*z*_/4, a non-zero *α*_*y*_ component of the unit magnetization vector appears, which has different signs in the left and right half of the nanoparticle. The appearance of the *α*_*y*_ component outside the diametral plane is associated with the vortex character of the domain walls.

Figure 4c,d show the distribution of magnetization in the same nanoparticle for three domain state with oppositely magnetized exchange cores of the vortex domain walls. As Fig. 4c shows, in the center of the left domain wall the component *α*_*x*_ = 1, whereas in the center of the right domain wall *α*_*x*_ = −1. Otherwise, the magnetization distributions for both three domain states are very close.

### fcc cobalt

Ii is well known^39,40,42,43^ that in addition to hcp cobalt nanoparticles, cobalt nanoparticles with fcc crystal structure can also be found in the experiment. For completeness, we calculate single-domain diameters for cobalt nanoparticles with fcc crystal structure having cubic type of magnetic anisotropy. In the calculations the cubic anisotropy constant was taken to be *K*_c_ = 2.7 × 10^6^ erg/cm^3^^40,42^, the particle saturation magnetization and the exchange constant being the same as for cobalt hcp nanoparticle.

**Figure 5:**
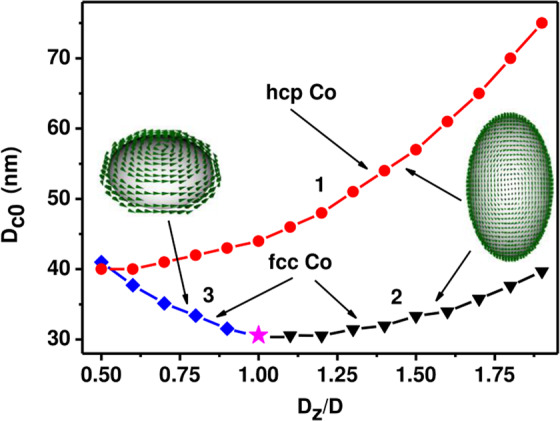
Single domain diameters for hcp and fcc cobalt nanoparticles depending on the particle aspect ratio: 1) cobalt hcp nanoparticles, (2) elongated cobalt fcc nanoparticles, *D*_*z*_/*D* > 1, 3) oblate cobalt fcc nanoparticles, *D*_*z*_/*D* < 1. Inserts show the direction of the vortex axis for the lowest inhomogeneous state in hcp and fcc type nanoparticles.

Figure 5 compares numerically calculated single domain diameters for cobalt nanoparticles with hcp and fcc crystal structures. The single domain diameters, *D*_*c*0_, shown in Fig. 5, correspond to the transverse particle diameter. It is easy to see in Fig. 5 that the single domain diameter for cobalt hcp nanoparticles significantly exceeds the one for fcc nanoparticle with the same aspect ratio. This is due to the increased value of the uniaxial magnetic anisotropy constant for cobalt hcp nanoparticles. As noted above, for cobalt hcp nanoparticles the transverse vortex is the lowest energy inhomogeneous state in the investigated range of particle aspect ratios. On the other hand, for oblate cobalt fcc nanoparticles with aspect ratio *D*_*z*_/*D* < 1, the axis of the vortex is parallel to the particle axis of symmetry (Z axis). However, for elongated cobalt fcc nanoparticles, *D*_*z*_/*D* > 1, the transverse vortex is energetically favorable.

## Discussion and conclusions

**Figure 6:**
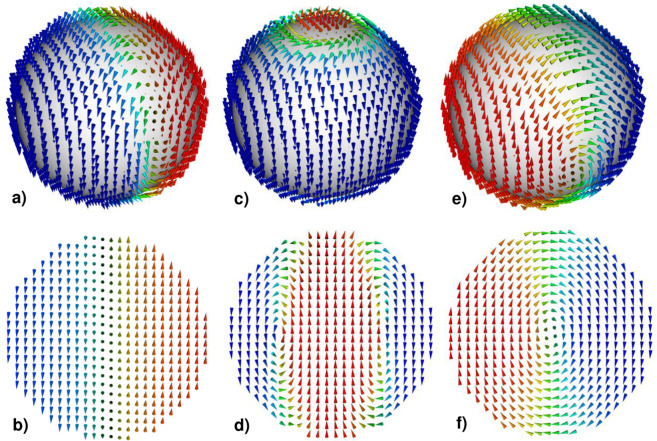
Various types of inhomogeneous micromagnetic states in a cobalt hcp nanoparticle with a uniaxial type of magnetic anisotropy: (**a,b**) Stapper’s two domain micromagnetic state^19^; (**c,d**) the cylindrical magnetic domain of Aharoni and Jakubovics^20^; (**e,f**) transverse vortex with the exchange core perpendicular to the particle easy anisotropy axis. (**a,c,e**) overall view of magnetization distributions, (**b,d,f**) are the corresponding diametral cross sections.

Numerical calculations of inhomogeneous magnetization distributions in spherical and ellipsoidal magnetic nanoparticles have a rather long history^2,19,20^. These calculations are of fundamental importance for Micromagnetics^4,5^ since such important quantity as the nanoparticle single-domain diameter is determined^4,5,10^ from a comparison of the total energy of a uniform magnetization with the energy of the lowest inhomogeneous micromagnetic state. In the pioneering work of Stapper^19^ the structure of a flat Bloch domain wall in a spherical cobalt nanoparticle with uniaxial magnetic anisotropy was calculated. The two domain state of Stapper is shown schematically in Fig. 6a,b under the assumption that the particle easy anisotropy axis is parallel to the Z axis. Based on his calculations, Stapper determined the single-domain diameter of the cobalt hcp nanoparticle as *D*_*с*0_ = 76.0 nm.

However, Aharoni and Jakubovics questioned^20^ the existence of plane domain structures constructed by Stapper, since the two-domain and three-domain micromagnetic states of Stapper break the initial axial symmetry of a spherical nanoparticle with uniaxial magnetic anisotropy. They proposed^20,21^ a 2D scheme of micromagnetic calculation, in which the distribution of magnetization in a spherical nanoparticle was approximated by small numerical cells of a toroidal shape. As the lowest energy inhomogeneous micromagnetic state they considered the structure of an axially symmetric cylindrical magnetic domain, shown schematically in Fig. 6c,d. For a nanoparticle with parameters of hcp cobalt, Aharoni and Jakubovics^20^ found this structure to be energetically favorable in comparison with the two domain state of Stapper, Fig. 6a. Accordingly, they obtained the value *D*_*с*0_ = 68.2 nm for the single-domain diameter of the hcp cobalt spherical nanoparticle, smaller than that of Stapper^19^.

Unfortunately, both 1D^19^ and 2D [20.21] micromagnetic calculations significantly limit the possible types of inhomogeneous micromagnetic distributions allowable in three-dimensional magnetic nanoparticle. In fact, it can be shown that trial magnetization distributions shown in Fig. 6(a–d) are unstable. 3D micromagnetic simulations^25–37^ showed that in magnetic nanoparticles with a small and moderate value of the anisotropy constant the vortex is the lowest energy inhomogeneous micromagnetic state. In a magnetically soft spherical nanoparticle, *K* ≪ *M*_*s*_^2^, with a diameter slightly exceeding the single domain one, the vortex arises as a result of instability of a uniformly magnetized state. As a result, the axis of the vortex is parallel to the easy anisotropy axis. This configuration can be stable in a certain range of diameters near the single domain diameter^38^.

However, with increase in the particle diameter the reduced radius of the exchange vortex core decreases and the rotation of the vortex axis into a plane perpendicular to the easy anisotropy axis turns out to be energetically favorable. As noted above, this process leads to a decrease in the particle magnetic anisotropy energy. On the other hand, for particles with a moderate value of the uniaxial anisotropy constant, *K* ~ *M*_*s*_^2^, only the transverse vortex state is stable. As shown in this paper, this is true, in particular, for cobalt hcp nanoparticles. The transverse vortex state in hcp cobalt nanoparticle is shown in Fig. 6e,f. For cobalt hcp nanoparticle it is precisely the transverse vortex state that competes in energy with a uniform magnetization. The true single domain diameter of spherical cobalt hcp nanoparticle is *D*_*с*0_ = 43.6 nm, that is, much smaller than that for cylindrical magnetic domain^20^.

In this paper, inhomogeneous magnetization distributions in spheroidal cobalt nanoparticles are obtained in a wide range of particle sizes based on the solution of dynamic Landau – Lifshitz – Gilbert equation. The complex vortex structure of domain walls separating two and three domain micromagnetic configurations is studied. It is shown that in cobalt hcp particles there are two types of three domain states with close total energy but different directions of magnetization of the exchange cores of the vortex domain walls. The domain wall width is calculated as a function of the transverse particle diameter. The areas of existence of three domain states in cobalt hcp nanoparticles with different aspect ratios are obtained. The remanent magnetizations of cobalt nanoparticles in multi-domain states are calculated.

For completeness, single-domain diameters are also calculated for cobalt fcc nanoparticles with cubic type of magnetic crystallographic anisotropy. It is shown that the single domain diameter for cobalt hcp nanoparticle significantly exceeds the corresponding value for cobalt fcc particle with the same aspect ratio. It seems that the numerical results obtained will be useful in interpreting experimentally observed domain structures in submicron-sized magnetic cobalt nanoparticles.

## Methods

Dynamics of the unit magnetization vector $\alpha (\overrightarrow{r})$ of a non single domain nanoparticle is described by the LLG equation^25,36^

1 $$\frac{\partial \overrightarrow{\alpha }}{\partial t}=-\,\gamma (\overrightarrow{\alpha }\times {\overrightarrow{H}}_{ef})+\kappa \left(\overrightarrow{\alpha }\times \frac{\partial \overrightarrow{\alpha }}{\partial t}\right),$$

where *γ* is the gyromagnetic ratio and *κ* is the phenomenological damping constant. The effective magnetic field ${\overrightarrow{H}}_{ef}$ acting on the unit magnetization vector can be calculated as a derivative of the total nanoparticle energy^36^

2 $${\overrightarrow{H}}_{ef}=-\frac{\partial W}{V{M}_{s}\partial \overrightarrow{\alpha }},\,{M}_{s}{\overrightarrow{H}}_{ef}=C\Delta \overrightarrow{\alpha }-\frac{\partial {w}_{a}}{\partial \overrightarrow{\alpha }}+{M}_{s}\overrightarrow{H}{\prime} $$

Here *V* is the nanoparticle volume, *M*_*s*_ is the saturation magnetization, *C* is the exchange constant, and $\overrightarrow{H}{\prime}$ is the demagnetizing field. The magneto-crystalline anisotropy energy density of hcp Co nanoparticle with uniaxial anisotropy is given by^36^

3 $${w}_{a}=K({\alpha }_{x}^{2}+{\alpha }_{y}^{2})$$

whereas for fcc Co nanoparticles with is the cubic anisotropy we use

4 $${w}_{a}={K}_{c}({\alpha }_{x}^{2}{\alpha }_{y}^{2}+{\alpha }_{x}^{2}{\alpha }_{z}^{2}+{\alpha }_{y}^{2}{\alpha }_{z}^{2})$$

here *K* and *K*_*c*_ are the uniaxil and cubic anisotropy constants, respectively.

For numerical simulation a non single-domain nanoparticle is approximated by a set of small ferromagnetic cubes of side *b* much smaller than the exchange length ${L}_{ex}=\sqrt{C}/{M}_{s}$ of the ferromagnetic material. Typically, several thousands of numerical cells, *N* ~ 10^3^–10^4^, is necessary to approximate with sufficient accuracy the vortex type magnetization distribution in nanoparticle volume. In the present calculations the magnetic damping parameter is given by *κ* = 0.5.

To find all stable micromagnetic states existing in a nanoparticle with given sizes and magnetic parameters it is necessary to carry our different runs of numerical simulations starting from various initial magnetization distributions. To save the time of numerical simulation in some cases one can use initial trial magnetization distributions resemble those expected for final stable micromagnetic configurations. In accordance with the Eq. (1), the final magnetization state is assumed to be stable under the condition

5 $${\max }_{(1\le i\le N)}|[{\overrightarrow{\alpha }}_{i}\times {\overrightarrow{H}}_{ef,i}/\Vert {\overrightarrow{H}}_{ef,i}\Vert ]| < {10}^{-6}$$

where ${\overrightarrow{\alpha }}_{i}$ and ${\overrightarrow{H}}_{ef,i}$ are the unit magnetization vector and effective magnetic field in the *i*-th numerical cell, respectively.
